# Outcomes of 23-gauge pars plana vitrectomy combined with phacoemulsification and capsulotomy without intraocular lens implantation in rhegmatogenous retinal detachment associated with choroidal detachment

**DOI:** 10.1097/MD.0000000000007869

**Published:** 2017-08-25

**Authors:** Huiyan Xu, David Lutrin, Zhifeng Wu

**Affiliations:** a Department of Ophthalmology, Nanjing Medical University Affiliated Wuxi Second Hospital, Wuxi, Jiangsu Province, China; b University of California, San Francisco, CA.

**Keywords:** 23-gauge pars plana vitrectomy, capsulotomy, choroidal detachment, IOL implantation, rhegmatogenous retinal detachment

## Abstract

Rhegmatogenous retinal detachment associated with choroidal detachment (RRDCD) is a special type of complex retinal detachment, and usually has a poor prognosis. This study aimed to assess the anatomical outcomes of 23-gauge pars plana vitrectomy (23G PPV) combined with phacoemulsification (phaco) and capsulotomy without intraocular lens (IOL) implantation in patients with RRDCD.

Seventy-six consecutive patients with RRDCD, who underwent retinal repair surgery from January 2010 to December 2014, were retrospectively analyzed. Forty patients underwent 23G PPV + phaco + IOL implantation, and 36 underwent 23G PPV + phaco + capsulotomy without IOL implantation (i.e., aphakia). All cases were filled with silicone oil. The follow-up time was 6 months after silicone oil was removed. Multivariate logistic regression analysis was the statistical method used.

The overall retinal anatomical reattachment rate was 58% (44/76): 40% (16/40) of patients receiving 23G PPV + phaco + IOL implantation; and 78% (28/36) of patients receiving 23G PPV + phaco + capsulotomy + aphakia (*P* = .007).

Surgical repair using 23G PPV + phaco + capsulotomy without IOL implantation can improve anatomical reattachment rates in patients with RRDCD.

## 1 Introduction

The incidence rate of rhegmatogenous retinal detachment (RRD) associated with choroidal detachment (RRDCD) is reported to be 2.0% to 4.5%.^[1–3]^ The anatomical reattachment rate after surgical repair in this type of retinal detachment, however, is usually low. Scleral buckling alone has been reported in 35% to 62% of cases.^[1–3]^ Primary vitrectomy results in a higher rate of retinal reattachment, reported to be as high as 77%.^[4–6]^ Studies have been performed to investigate the outcomes of vitrectomy combined with other surgical techniques. It is reported that pars plana vitrectomy (PPV) combined with scleral buckling improved the reattachment rate in RRDCD.^[7,8]^ By contrast, however, a European multicenter retrospective study suggested that a supplemental buckle may not be helpful in RRDCD.^[9]^ Some surgeons suggested routine lensectomy, or intraocular lens (IOL) removal and capsulotomy in complex retinal detachment.^[10,11]^ Nevertheless, few studies investigating capsulotomy in patients with RRDCD have been performed. Accordingly, we retrospectively observed 76 Chinese patients with RRDCD in our hospital from 2010 to 2014, and analyzed the efficacy of 23-gauge (23G) PPV (23G PPV) combined with phacoemulsification (phaco) and capsulotomy without IOL implantation on anatomical retinal reattachment rates.

## 2 Methods

A retrospective review including 76 patients who met the following inclusion criteria was performed: RRD with identification of breaks; presence of choroidal detachment (CD) diagnosed preoperatively using ocular ultrasound; proliferative vitreoretinopathy (PVR) with grades (1983, United States) C or D (i.e., required vitrectomy surgery); operation(s) performed by 1 surgeon; and silicone tamponade. Patients who experienced trauma, tumor(s), or exudative disease, or who underwent previous vitrectomy or phaco were excluded. The study was approved by the hospital's ethics committee.

All cases received the same pre- and postoperative treatments. A sclerotomy, through which the choroidal fluid had drained, was performed and subsequently re-entered using a 23G blade to penetrate the pars plana canals. 23G PPV + phaco + IOL implantation, or 23G PPV + phaco + capsulotomy without IOL implantation (i.e., aphakia), was performed. Membrane peeling or retinectomy was performed if needed. Iridotomy was performed before silicone oil tamponade in cases with aphakia. All cases were treated with intraocular laser around retinal breaks and filled with silicone oil. All patients were followed-up for 6 months after silicone oil removal. Unsuccessful cases included: failure of retinal detachment repair; remaining silicone oil at study conclusion; or need for additional procedures to repair detachments.

Multivariate logistic regression analysis (Tables 1 and 2), chi-squared test, and ANOVA test (Table 3) were performed using SPSS version 19.0 (IBM Corporation, Armonk, NY); *P* < .05 was considered to be statistically significant.

**Table 1:**
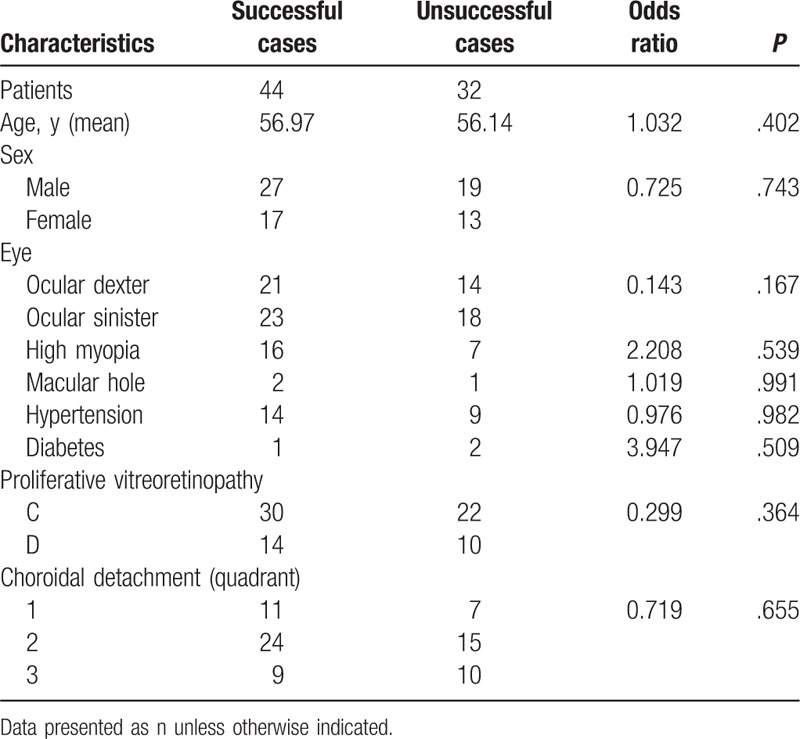
Comparison of basic demographic information between successful and unsuccessful cases before operation.

**Table 2:**
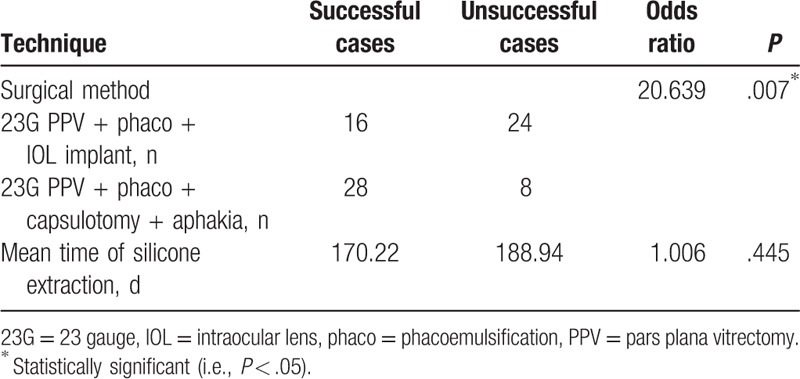
Comparison of surgical techniques between successful and unsuccessful cases.

**Table 3:**
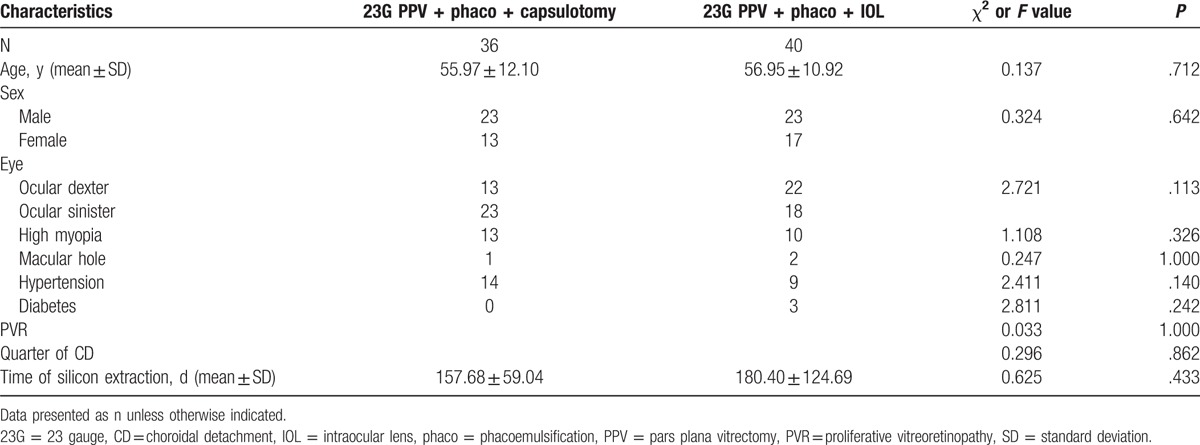
Comparison of characteristics between patients treated with different methods of surgery.

## 3 Results

### 3.1 Demographic data

Seventy-six eyes of 76 patients met the inclusion criteria. The mean age of the patients was 56 years (range 17–78 years). Sixteen patients were high myopia, and 2 had a macular hole. Basic demographic information is summarized in Table 1.

### 3.2 Outcomes

The overall retinal anatomical reattachment rate was 58%. Forty patients underwent 23G PPV + phaco + IOL, with a reattachment rate of 40% (16/40); 36 received 23G PPV + phaco + capsulotomy + aphakia, with a reattachment rate of 78% (28/36) (*P* = .007) (Table 2). Of the unsuccessful cases, 20 patients underwent additional surgeries, 5 of whom eventually achieved retinal reattachment.

The basic information and analysis of patients who underwent the different surgeries are listed in Table 3.

## 4 Discussion

RRDCD is a special type of RRD. Hypotony, macular hole, longer axial length, and whole retinal detachment maybe potential risk factors for the development of CD in RRD patients in the Chinese population.^[12]^ This type of retinal detachment has a rapid progression and a higher recurrence rate, resulting in poor prognosis.^[9,10]^ Primary vitrectomy has recently been proposed as the first-line treatment choice for such special retinal detachments.^[11]^ Using this surgical technique, proliferative and concentrated vitreous can be excised, and all retinal holes can be identified.^[6–8]^ However, the detachment recurrence rate remains high when vitrectomy is performed alone. Several studies investigating methods to improve anatomical outcomes have been performed. Some have proposed that vitrectomy combined with scleral buckling may improve the reattachment rate,^[7,8]^ while others suggested that vitrectomy combined with lensectomy without IOL implantation maybe better in cases involving complex retinal detachment.^[13,14]^ On review of these investigations, however, no clear consensus regarding optimal treatment has been reached.^[5–8]^

Our study compared the anatomical outcomes of 23G PPV + phaco + IOL implantation versus 23G PPV + phaco + capsulotomy without IOL implantation in RRDCD. We found that 23G PPV + phaco + capsulotomy without IOL implantation had a significantly higher reattachment rate (78%) than 23G PPV + phaco + IOL implantation (40%).

Retinal detachment recurrence is primarily due to PVR.^[11]^ In contrast to patients with RRD, intravitreous inflammatory mediators are upregulated in patients with RRDCD, who usually experience more severe PVR.^[15]^ The overall retinal anatomical reattachment rate after the first primary vitrectomy in our study was only 58%, which was low. However, the 23G PPV + phaco + capsulotomy without IOL implantation had a higher reattachment rate, which was approaching to rates previously reported in the literature.^[4–6]^ Vitrectomy and capsulotomy may improve anterior and base vitrectomy, which may decrease anterior PVR.^[13,14]^ A study by Tseng et al reported that pseudophakic eyes exhibited a higher proportion of hypotony after retinal reattachment surgery than eyes without IOL implant.^[16]^ The lens capsule may fibrose and contract, causing secondary contraction of the ciliary body, which in turn leads to chronic hypotony.^[17,18]^ Because RRDCD is usually accompanied by hypotony, possible advantages of capsulotomy include a lower hypotony rate after surgery, which may improve the reattachment rate in RRDCD. And the results of our study support the points.

Our study had several limitations. No further retinal reattachment rates were assessed after successive surgeries because some patients who experienced retinal detachment recurrence refused additional treatment. Visual outcomes—which may be as important as anatomical outcomes—were not assessed. Further prospective studies involving patients with RRDCD are required.

## 5 Conclusions

Surgical repair using 23G PPV + phaco + capsulotomy without IOL implantation can improve anatomical reattachment rates in patients who experience RRDCD.
